# Quantifying Levels of Peste Des Petits Ruminants (PPR) Virus in Excretions from Experimentally Infected Goats and Its Importance for Nascent PPR Eradication Programme

**DOI:** 10.3390/v11030249

**Published:** 2019-03-12

**Authors:** Satya Parida, M. Selvaraj, S. Gubbins, R. Pope, A. Banyard, Mana Mahapatra

**Affiliations:** 1 The Pirbright Institute, Ash Road, Woking, Surrey GU24 0NF, UK; Muni.NarayananSelvaraj@pirbright.ac.uk (M.S.); simon.gubbins@pirbright.ac.uk (S.G.); mitaliparida@yahoo.co.uk (R.P.); mana.mahapatra@pirbright.ac.uk (M.M.); 2 Animal and Plant Health Agency, Weybridge, Surrey KT15 3NB, UK; ashley.banyard@apha.gov.uk

**Keywords:** peste des petits ruminants, morbilliviruses, viral nucleic acid detection, body excretions, real-time RT-PCR

## Abstract

Following the successful eradication of rinderpest, the World Organization of Animal Health (OIE) and the Food and Agriculture Organisation (FAO) have set a goal to globally eradicate Peste des petits ruminants (PPR) by 2030. To support the eradication programme we have quantified the levels of PPR virus (PPRV) nucleic acid excreted in body fluids (blood, feces, saliva, nasal and eye swabs) of PPRV-infected goats to ascertain which days post-infection animals are potentially infectious, and hence direct quarantine activities. The data will also indicate optimal sample strategies to assess presence of PPR infection in the naturally infected herd. Peak PPRV nucleic acid detection in different bodily fluids was between 5 and 10 days post-infection. As such, this period must be considered the most infectious period for contact transmission, although high viral load was observed through RNA detection in nasal excretions from two days post-infection until at least two weeks post-infection. Percentage sample positivity was low both in eye swabs and saliva samples during the early stage of infection although RNA was detected as late as two weeks post-infection. From the individual animal data, PPRV was detected later post-infection in fecal material than in other body fluids and the detection was intermittent. The results from this study indicate that nasal swabs are the most appropriate to sample when considering molecular diagnosis of PPRV.

## 1. Introduction

Peste des petits ruminants (PPR), places a huge disease burden on agriculture across the developing world, in particular affecting small ruminant production and in turn increasing poverty in many developing countries [1,2]. PPR virus (PPRV) is classified as a distinct virus species, termed small ruminant morbillivirus, within the *Morbillivirus* genus, family Paramyxoviridae, alongside *Measles morbillivirus* (MV), *Rinderpest morbillivirus* (RPV), *Canine morbillivirus* (CDV) [3], *Phocine morbillivirus* (PDV), *Porpoise morbillivirus* (PMV), *Dolphin morbillivirus* (DMV) [4,5] and *Feline morbillivirus* (FeMV) [6]. Evidence for a number of other novel morbilliviruses has been reported in bats and rodents, although live virus has not been isolated to date [7]. PPRV has a non-segmented single-stranded negative sense RNA genome that contains six contiguous, non-overlapping transcription units that encode six structural proteins: the nucleocapsid protein (N); the phosphoprotein (P); the matrix protein (M); the fusion protein (F); the haemagglutinin protein (H) and the large (L) polymerase protein and two non-structural proteins (V and C protein) that are encoded from the P-gene transcription unit using overlapping open reading frames (ORFs) and co-transcriptional editing to access the third ORF to generate C [8]. PPRV exists serologically as a single serotype but is genetically divided into four distinct lineages (I, II, III and IV).

The highly contagious nature of PPRV and the distribution and movement of small ruminants creates a serious trans-boundary problem, inhibiting trade and heightening economic losses in affected areas where small ruminants are often more important than cattle in food production [1]. PPR was long considered to be confined to West Africa but later it was described throughout Africa (except some southern African countries) from south of the Sahara to north of the Equator, as well as in the Middle East and Asia [1,2]. Historically, Europe has been free of PPR, though that changed following an incursion into Bulgaria during 2018 [9]. Following the successful eradication of rinderpest, the World Organization of Animal Health (OIE) and the Food and Agriculture Organisation (FAO) have set as a target the global eradication of PPR by 2030. The initial phase of any eradication programme requires an efficacious vaccine accompanied by robust diagnostic tools to enable detection of viral products in appropriate biological samples collected at appropriate times during infection. 

PPRV is both lympho- and epitheliotropic and typical signs of infection include pyrexia, conjunctivitis, rhinotracheitis, ulcerative stomatitis, gastroenteritis and in severe cases pneumonia. Pathology is generally driven by opportunistic infection following the profound leucopenia caused by PPR infection of lymphocytes. Morbidity and mortality rates can be 90%–100% in naïve populations, dropping to nearer 20% in endemic areas [1]. PPRV is mainly transmitted by droplet spread from respiratory discharges and, as such, often requires close contact between animals to spread within a herd [1,10]. In addition to the droplet spread between animals in close contact, other transmission routes are also thought possible. Excretion of virus in different bodily fluids has been documented from experimental animals [11,12], however very little information is available on the routes and duration of virus excretion; such data, where it exists, is usually fragmentary or qualitative [13,14,15,16]. In this study we have quantified viral shedding in various bodily excretions (blood, feces, saliva, nasal and eye swabs) of goats intranasally infected (simulating natural infection) with two (out of the four) lineages of PPRV. This information is a useful guide to determining optimal sampling days post infection that in turn can help in (i) optimising diagnosis of the disease by collecting appropriate samples at appropriate times, and (ii) to indicate potential quarantine periods following suspicion of disease within a herd that may help better control the spread of the disease.

## 2. Materials and Methods

### 2.1. Ethics Statement

Animal experiments were conducted according to the UK Home Office regulations (Project licence number: 70/6907) and after approval by The Pirbright Institute (TPI) Animal Welfare and Ethical Review Board (AWERB), Pirbright, UK. The recommendation of the review board was strictly followed.

### 2.2. Goat Infection Study

Animal experiments were conducted at the animal units at TPI, United Kingdom. The animals sampled for this study were challenge controls for two PPR vaccine studies (*n* = 5/group) carried out during 2017. European mixed breeds of apparently healthy male goats, aged 6–9 months were used in these studies. Animals in each study were kept for at least seven days to acclimatize them to the laboratory environment prior to experimental infection with PPRV. During that time, all the animals were found to be healthy and free of disease, with normal rectal temperatures ranging from 38.5–39.1 °C. Animals in each group were challenged with 10^5^ TCID_50_ virulent PPRV (Lineage II: Ghana/78 (LK1/V1/VDS1) and Lineage IV: Morocco/2008 (PLN1/VDS3) by the intranasal route using the LMA^®^ MAD Nasal™ Intranasal Mucosal Atomization Device (LMA, San Diego, CA, USA) as described previously [12] to mimic natural infection. Animals were observed at least twice daily with recording of clinical signs and rectal temperature for up to 14 days. The animals in both the trials were treated with antibiotics to avoid secondary infection, and the animals that developed severe clinical signs were humanely euthanized according to an established clinical scorecard [17]. Ocular, nasal and oral swabs, EDTA blood and fecal samples were collected pre-challenge (day 0), and every day or every alternate day post-challenge (dpc). Fecal samples were collected from the floor of the animal rooms both for individual animals, as well as on occasion for pooled samples. All the samples were stored at −70 °C until processed.

### 2.3. Molecular Assessment of Blood, Swabs and Fecal Samples

Nasal, mouth and eye swabs were collected in 130 µL of lysis buffer (MagMAX-96™ Viral RNA Isolation Kit, Thermo Fisher Scientific, Vilnius, Lithuania) and stored at −80 °C until RNA extraction. Total RNA was extracted from the swab samples using the KingFisher Flex automated extraction platform (Thermo Fisher Scientific, UK) with the MagMAX-96™ viral RNA isolation kit (Thermo Fisher Scientific, UK) following the manufacturer’s protocols and the RNA eluted in a final volume of 90 µL. RNA extraction from blood was carried out using Trizol (Invitrogen, Carlsbad, CA, USA) following the manufacturer’s protocol. Briefly, 200 μL of blood was mixed with 800 μL of Trizol reagent and the RNA eluted in a final volume of 40 μL. Total RNA was extracted from the fecal samples following the method as described previously [18]. For fecal material, where solid pellets were present approximately 1 g of fecal matter was homogenised in 3 mL of M25 buffer using a mortar and pestle; for loose fecal samples, approximately 1 mL of material was diluted in 3 mL of M25 buffer as above and any solid fragments triturated with a mortar and pestle. In addition, the eluted RNA from fecal samples was further purified using the RNeasy mini RNA extraction Kit (Qiagen, Hilden, Germany) to remove polymerase chain reaction (PCR) inhibitors present in fecal material following the manufacturer’s protocol after dilution to 100 µL in nuclease free water. The final RNA elution volume was 40 μL. All RNA samples were stored at −70 °C as single use aliquots until tested.

Reverse transcription-polymerase chain reaction (RT-PCR) was carried out to amplify the N-terminus of the N-gene only using the RNA extracted from the faecal matter as previously described [19] using the primer sets same as in real-time RT-PCR (qRT-PCR). All ocular, nasal and mouth swabs, blood and faecal samples were analyzed by qRT-PCR to assess the viral load [20] using Superscript III Platinum R one step qRT-PCR system kit (Invitrogen) on the ABI 7500 system (Applied Biosystems, Foster city, CA, USA). All samples were run in duplicate and the samples showing positive in only one well were repeat-tested for confirmation.

### 2.4. Construction of Plasmid for RNA Standard

The PPR vaccine strain, Nigeria 75/1, was grown on Vero cells using standard methods and total RNA was extracted using Trizol following the manufacturer’s protocol. This RNA was used to amplify the viral gene (N-gene) for construction of the plasmid to generate RNA standards. The RT-PCR target region in the N-gene of PPR vaccine strain Nigeria 75/1 was amplified using gene specific primer sets as described [20], and cloned under the control of the T7 promoter in the pGEM3z vector (Promega, Maddison, USA). The plasmid was sequenced on both the strands to ensure no nucleotide changes had been introduced during the cloning process. The plasmid was linearlized using *Sal I* (New England Biolabs Ltd., Ipswich, MA, USA) and in vitro transcription was carried out using T7 Megascript kit (Invitrogen, Vilnius, Lithuania). The in vitro transcribed RNA was treated with DNase for complete elimination of plasmid DNA from the transcripts. The RNA was purified using a Megaclear transcription clean-up kit (Ambion, Austin, USA) followed by quantification using a spectrophotometer. A 10-fold serial dilution of the transcript preparation (10^8^–10^1^  copies) was prepared in RNAse-free water in single use aliquots and stored at −70 °C until use. These standards were used in triplicate for the quantification of copies as a standard curve assay. 

### 2.5. Statistical Analysis

Virus levels in goats infected with two strains of PPRV were compared for four of the sample types (blood and eye, nasal and oral swabs) by fitting the following curve,

(1) $$y(t)=\alpha (\frac{t}{\beta }{)}^{\beta^{2}/\gamma^{2}}\exp\left(\frac{-\beta (t-\beta )}{\gamma^{2}}\right),$$

to data for individual goats. Here *y*(*t*) is the level of virus at *t* days post-challenge (dpc), *α* is the peak level of viral RNA (log_10_ RNA copies/mL), *β* is the time at which the peak level was observed, and *γ* is the time before (and after) the peak at which the maximum rate of change in shedding occurs. The parameters (*α*, *β* and *γ*) were allowed to vary among sample types and PPRV strains (fixed effect) and among animals (random effect). Analyses were implemented using the nlme package [21] in R (version 3.5.1) [22].

In addition, the proportion of positive samples for these sample types was compared using a generalised linear mixed model with binomial errors and a logit link function. The response was whether or not a sample was positive for viral RNA, with days post-challenge (as a quadratic function), sample type and strain as fixed effects and animal as a random effect. The analysis was implemented using the MASS package [23] in R (version 3.5.1) [22].

For detection of PPRV RNA in feces, a descriptive analysis was carried out because of the smaller number and intermittent nature of positive samples. In addition, fecal samples for the Morocco strain were taken from individual animals, while those for the Ghana strain were from pools (i.e., multiple animals).

## 3. Results

**Figure 1:**
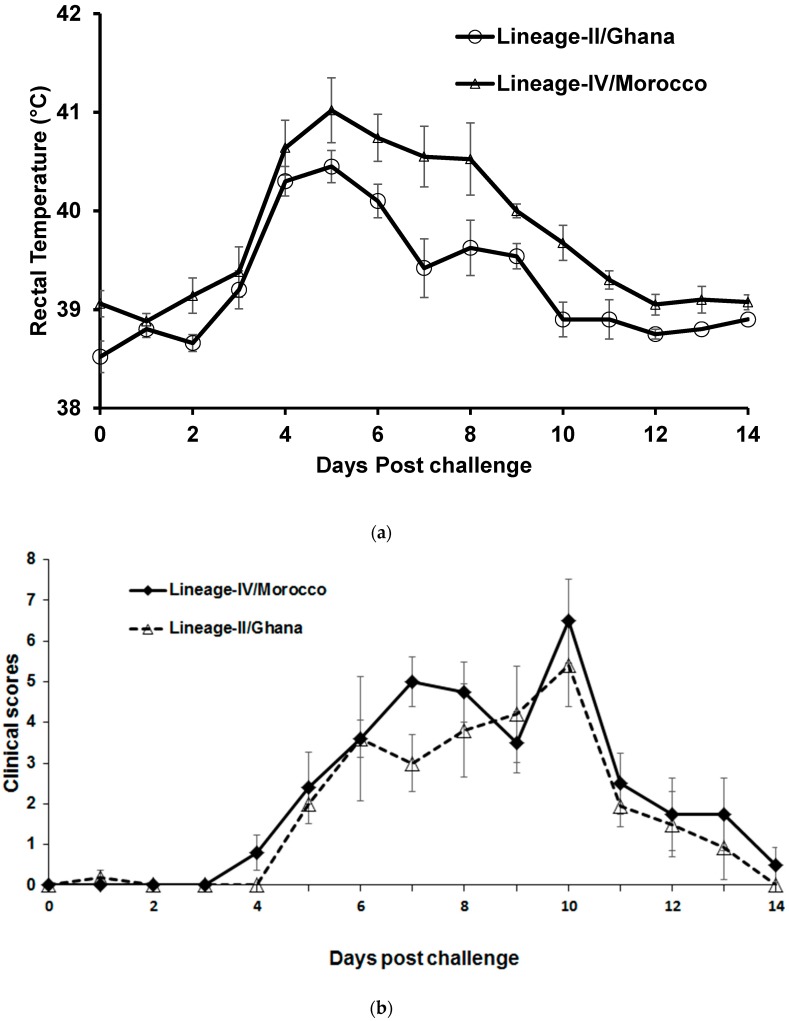

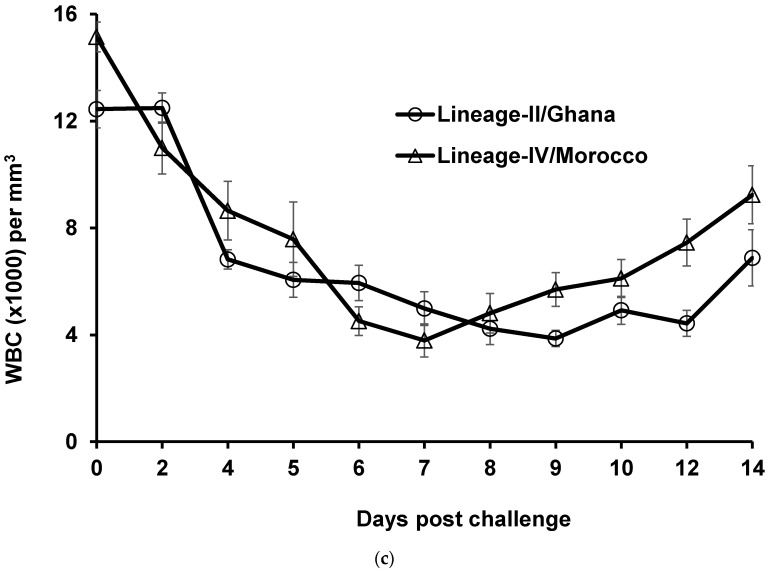
(**a**) Rectal body temperature of goats post-challenge; (**b**) Mean clinical score of Peste des petits ruminants virus (PPRV)-challenged goats; (**c**) Mean leucocyte count in blood of PPRV-challenged goats.

During the first week after arrival (i.e., before challenge) animals showed a rectal temperature of 38.5 to 39.1 °C. Following intranasal challenge the animals were clinically infected with an increase in body temperature, reaching more than 40 °C for all animals during the study, with those infected with the Moroccan isolate reaching a peak pyrexia of >41 °C on day 5 post-challenge (Figure 1a). Of the two different virus isolates used in this study, the infected animals were treated with antibiotics on 5 dpc onwards. Animals in both the groups exhibited a slight delay in the development of clinical disease with rectal temperatures rising at 3 dpc, reaching a peak pyrexia 5 dpc, after which temperatures started to decline, although some animals infected with the Moroccan lineage IV isolate remained pyrexic with temperatures exceeding 40 °C until 8 dpc (Figure 1a). The animals in both the groups showed comparable clinical scores (Figure 1b). Nasal discharge was observed in most animals at 3–4 dpc, which became muco-purulent by 5–6 dpc. There was congestion of nasal and buccal mucosae, ocular discharge and appearance of mouth/dental pad lesions from day 4–6, which persisted for a further 7 days, prior to convalescence. Some infected animals exhibited reduced feeding, indicating oral trauma, and mouth lesions were observed. Animals developed a pronounced leucopenia, post-challenge, with leucocyte counts reaching as low as ~4000 leucocytes/mm^3^ of blood by 7 dpc for Lineage IV (Morocco strain) and 9 dpc for Lineage II PPRV (Ghana strain), with a gradual recovery in leucocyte numbers observed after this point for those not terminated according to the clinical score sheet (Figure 1c). 

### Levels of Viral Nucleic Acid in Clinical Materials

**Figure 2:**
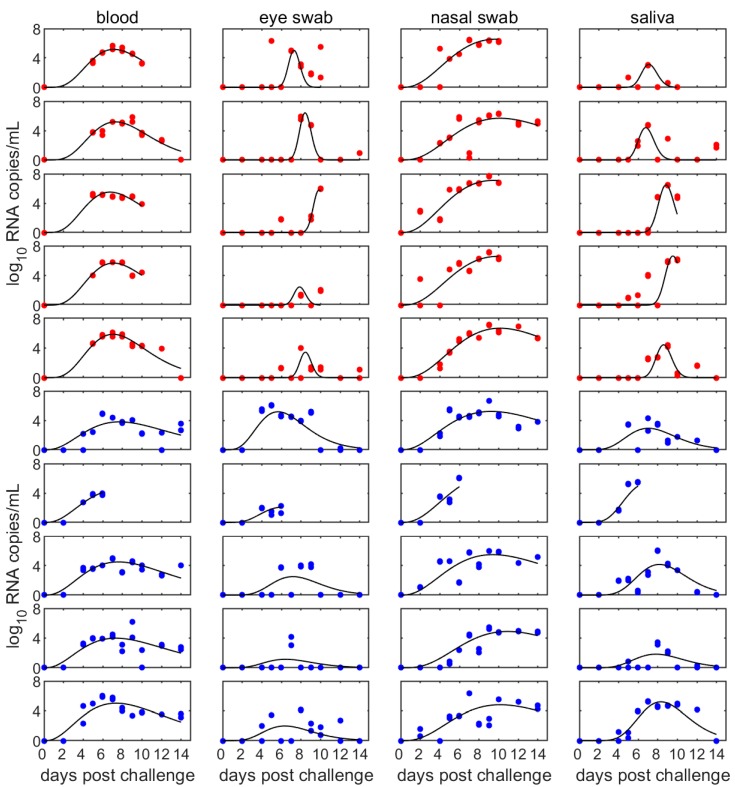
Levels (log_10_ RNA copies/mL) of PPRV RNA in different samples types: blood (first column); eye swabs (second column); nasal swabs (third column); or saliva (fourth column). Results are shown for ten goats infected with either the Lineage-II/Ghana (top five rows; red symbols) or Lineage-IV/Morocco (bottom five rows; blue symbols) strain of PPRV. The circles are the observed levels of viral RNA and the black line is the fitted curve for the animal.

The observed levels of viral RNA in four sample types (blood, eye swabs, nasal swabs and saliva) for goats infected with the Ghanain and Moroccan isolates are shown in Figure 2. The estimated parameters for the fitted curves (and their standard errors) are shown in Table S1. The mean peak virus level did not differ significantly (*p* > 0.05) among sample types or strains, except for eye swabs taken from goats infected with the Moroccan strain of PPRV. Rather, most differences in peak virus levels were ascribed to between-animal variation. By contrast, the timing of peak levels differed amongst strains and sample types. For goats infected with the Ghanaian strain, peak levels occurred earliest for blood (7.1 dpc), then saliva (8.2 dpc) and eye swabs (8.4 dpc), and last in nasal swabs (9.9 dpc), while for those infected with the Moroccan strain they were earliest in eye swabs (6.4 dpc), then blood (7.5 dpc) and saliva (7.7 dpc) and last in nasal swabs (9.9 dpc).

**Figure 3:**
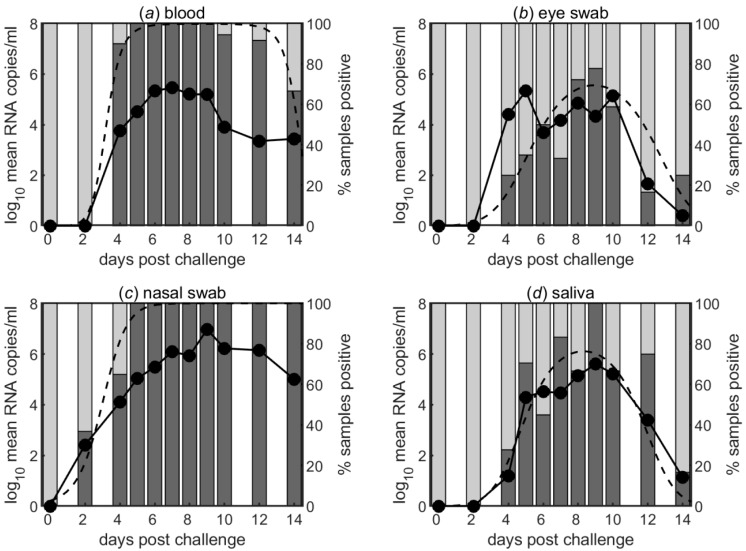
Mean levels of viral RNA (log_10_ mean RNA copies/mL) in (**a**) blood, (**b**) eye swabs, (**c**) nasal swabs and (**d**) saliva swabs taken at different days post-challenge from goats infected with PPRV (left axis). The bars indicate the percentage of samples that were positive at each day post-challenge and the black dashed line indicates the model for the proportion of positive samples (right axis).

Although the mean levels of viral RNA present in different body fluids (blood, eye, nasal and saliva swabs) did not differ greatly up to 10 dpc (Figure 3; Figure 2), the proportion of positive samples differed amongst the sample types (Figure 3; see also Table S2). In particular, the proportion of positive samples rises earliest and to higher levels for nasal swabs and blood compared with eye swabs and saliva. Furthermore, the proportion of positive samples declines to low levels after around 9 dpc for both eye swabs and saliva. This pattern in the proportion of positive samples in each sample type did not differ between the Ghanaian and Moroccan strains.

**Figure 4:**
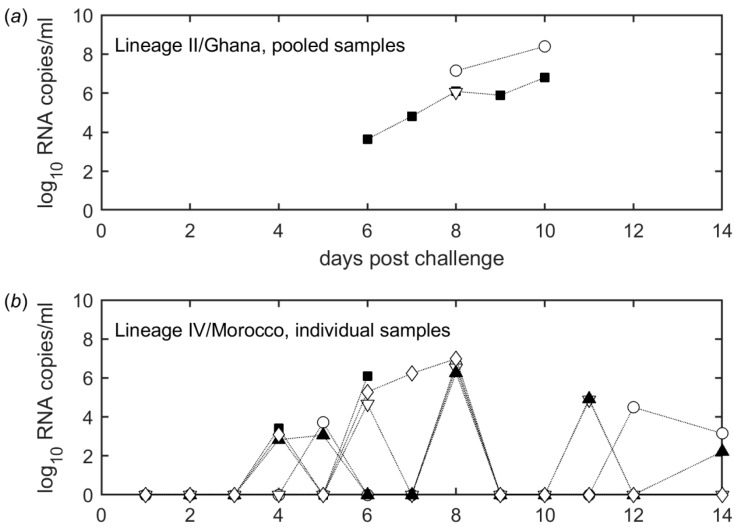
Detection of PPRV RNA in feces. (**a**) Pooled samples from goats infected with the Lineage II/Ghana strain. The same symbols indicate fecal samples taken from the same pool. (**b**) Samples from individual goats infected with the Lineage IV/Morocco strain. The same symbols indicate fecal samples taken from the same animal.

Viral RNA was also detected in feces for both strains and from 4 dpc in individual samples and from 6 dpc in pooled samples (Figure 4a,b). The animal-level data shows that PPRV nucleic acid was detected only intermittently in fecal matter from individuals (Figure 4b), but pooled samples were consistently positive for viral RNA (Figure 4a).

## 4. Discussion

PPR remains a significant threat to the establishment of sustainable agriculture in areas where the virus is present. Syndromic surveillance is fraught with potential differential diagnoses with numerous viral pathogens of small ruminants (e.g., bluetongue virus, caripox virus and foot-and-mouth disease virus) causing similar early disease signs to PPR. Furthermore, PPR can present in a variety of clinical manifestations with morbidity and mortality rates ranging from low to severe. The potential for mild PPR to circulate within herds makes syndromic surveillance very difficult with PPR only often being suspected following explosive epizootics in susceptible populations.

PPR is mainly transmitted through droplet spread via the respiratory route and, as such, close contact is often required for efficient transmission. However, virus and viral products have been described in excreta including the profuse lacrimation and mucopurulent discharges seen in the acute phase of the disease. The need to potentially sample animals to evaluate the presence or absence of PPR in a population requires that the kinetics of viral shedding in the key sample types are defined. Shedding of virus is also of high interest in natural infections as it relates directly to the transmissibility of the pathogen and, hence, can guide quarantine actions.

Here, challenge control animals from experimental studies have been sampled to assess the sites and timing of viral excretion with the aim of guiding field sampling for viral diagnosis in nascent eradication programme. In the present study, experimental doses of virus were sprayed into the nasal turbinate’s of naïve British white goats using a method that mimics natural infection. Sampling of goats at different time points post infection enabled a comprehensive analysis of viral RNA shedding in these animals, infected with two different lineages of PPRV, to be conducted. It is clear from these two studies that PPRV can be excreted from one to two days before or at the latest by the time of onset of viraemia, at least by the nasal route; infected goats may, therefore, contaminate the environment before the onset of viraemia similar to rinderpest infection in cattle [24]. Similarly, PPRV is demonstrated to be excreted 2–3 days before the appearance of clinical signs [25]. The peak period for PPRV RNA excretion in different body fluids was observed from 5 to 10 days post-challenge although a reasonable amount of virus excretion in 60% of infected samples was observed in the nasal excretions minimum up to two weeks post-infection (end day of this study). Therefore, to be on the safer side, a minimum three to four weeks’ quarantine period is advocated. This is supported well by previous work where one goat out of 12 PPRV-infected goats excreted virus up to 26 days post-infection [26].

There was no significant difference in clinical scores between the two groups (Figure 1b). Virulence factors associated with the severity of the disease have not been clearly defined for PPR. In fact, both viral and host factors involved in determining the outcome of infection with PPRV remain largely unknown. It is clear that the immunological and nutritional status of the infected animal is of importance in dictating survival, as is the presence of opportunistic pathogens within the infected animal that may drive clinical disease as an opportunistic infection in the face of profound leucopenia driven by early viral replication in lymphocytes. 

Evaluation of excretion of viral products was limited to molecular analyses because, although these methods do not detect live virus, previous studies have strongly correlated the detection of viral RNA in blood and ocular discharges with the shedding of live virus [11,12]. In addition, swabs and faecal samples from infected animals can be difficult to assess using virus isolation because of bacterial or fungal contamination of sterile tissue culture systems although robust use of antibiotics and anti-fungal agents are recommended. Importantly, RT-PCR is recognised as the most sensitive tool for the detection of viral products and, as such, was used as the focus of this study. The observation that viral RNA was detected most readily in, and for a more protracted time period that other samples, nasal swabs makes this sampling method the optimal non-invasive method for sampling animals suspected of having PPR. Certainly, the non-invasive nature of nasal swabbing makes it far more appropriate than blood sampling, the sample type with the second highest likelihood of viral nucleic acid detection. Whilst nasal swabbing was most productive in terms of detecting viral nucleic acid, sampling other fomites also demonstrated the presence of viral RNA in samples. Of interest, the detection of viral nucleic acid in fecal samples can be used as non-invasive samples, particularly in wildlife sampling [15,27,28,29,30], albeit at a low viral RNA copy number, raises questions about viral shedding in fecal matter and whether this represents a potential mechanism of virus transmission. Certainly further studies are warranted. 

## 5. Conclusions

In conclusion, the excretion of PPRV nucleic acid in different body fluids differs. Regardless, in the present study the peak period for PPRV RNA excretion in different body fluids was defined as being from 5 to 10 days post-challenge, although a high RNA load was observed in nasal excretions up to two weeks post-challenge. The lower detection of viral RNA in both eye swab and saliva samples at two weeks post-challenge suggest that these samples are suboptimal for viral diagnosis in the late phase of infection. However, these samples at the peak period of infection may be considered as less contaminated than nasal samples for the purpose of virus isolation. However, peripheral blood mononuclear cells (PBMCs) are the best samples for virus isolation in our hands. From the individual animal data, PPRV was detected later in feces than other body fluids, although detection of viral RNA in this sample matrix was intermittent, and as such is not ideal for molecular diagnosis. As both nasal swabs and EDTA blood samples were positive for viral nucleic acid at both early and late time points post-infection, they should be considered most suitable for the molecular diagnosis of PPRV. Considering the non-invasive sampling regime for domestic small ruminants in an outbreak situation in the field, nasal sampling may be considered optimal for molecular diagnosis over blood sampling. However, where resources allow, paired sample (nasal and blood) analysis may be recommended for molecular diagnosis of PPRV. Certainly, defining this aspect of sample acquisition and testing may aid in the overarching goal of global PPR eradication. 

## Supplementary Materials

The following are available online at https://www.mdpi.com/1999-4915/11/3/249/s1, Table S1: Peak level and timing of peak of peste des petits ruminants virus (PPRV) RNA in goats for different sample types and strains. Table S2: Temporal changes in the proportion of samples positive for peste des petits ruminants virus (PPRV) RNA in goats for different sample types and strains.
