# Supplementary material for: Quantifying Levels of Peste Des Petits Ruminants (PPR) Virus in Excretions from Experimentally Infected Goats and Its Importance for Nascent PPR Eradication Programme
Source: Viruses. 2019 Mar 12;11(3):249. doi: 10.3390/v11030249 (PMC6466160; doi:10.3390/v11030249)
Supplement: Supplementary file 1 [file viruses-11-00249-s001.pdf]

Table S1. Peak level and timing of peak of peste des petits ruminants virus (PPRV) RNA in goats for different sample types and strains.

parameter	fixed effects		random effects
	estimate†	standard error	animal‡
level of peak viral shedding ($\log_{10}$ RNA copies/ml; α)			
Ghana, blood (baseline)	5.48	0.61	
Ghana, eye swab	-0.84	1.02	
Ghana, nasal swab	1.04	0.86	
Ghana, saliva	-0.46	0.91	1.18
Morocco, blood	-1.13	0.86	
Morocco, eye swab	**-2.90**	0.87	
Morocco, nasal swab	-0.11	0.88	
Morocco, saliva	-1.59	0.88	
time of peak viral shedding (days post challenge; β)			
Ghana, blood (baseline)	7.07	0.42	
Ghana, eye swab	**1.30**	0.55	
Ghana, nasal swab	**2.80**	0.83	
Ghana, saliva	**1.10**	0.55	0.76
Morocco, blood	0.47	0.68	
Morocco, eye swab	-0.71	0.69	
Morocco, nasal swab	**2.81**	0.79	
Morocco, saliva	0.58	0.62	
time of maximum increase in viral shedding (days post challenge; γ)			
Ghana, blood (baseline)	3.10	0.33	
Ghana, eye swab	**-2.51**	0.35	
Ghana, nasal swab	**2.56**	0.88	
Ghana, saliva	**-2.30**	0.34	7.03×10^{-6}
Morocco, blood	**1.37**	0.60	
Morocco, eye swab	-0.65	0.47	
Morocco, nasal swab	**2.62**	0.86	
Morocco, saliva	-0.61	0.43	

† the mean parameter for each sample type and strain is the estimate for the baseline plus that for the specific sample type and strain; values shown in bold are significantly ($P<0.05$) different from zero

‡ standard deviation in parameter for between-animal variation; common to all sample types and strains

Table S2. Temporal changes in the proportion of samples positive for peste des petits ruminants virus (PPRV) RNA in goats for different sample types and strains.

parameter	estimate†	standard error
intercept		
blood (baseline)	-9.62	1.96
eye swab	2.51	2.25
nasal swab	6.02	2.17
saliva	1.81	2.26
linear term (dpc)		
blood (baseline)	3.66	0.61
eye swab	-1.88	0.68
nasal swab	-2.62	0.93
saliva	-1.50	0.69
quadratic term (dpc)		
blood (baseline)	-0.21	0.04
eye swab	0.11	0.04
nasal swab	0.27	0.12
saliva	0.09	0.04

† the coefficient for each sample type is the estimate for the baseline plus that for the specific sample type
